# Concurrent Extracerebral Vasoconstriction in Patients with Reversible Cerebral Vasoconstriction Syndrome: A Cross-Sectional Study

**DOI:** 10.3390/jcm14134402

**Published:** 2025-06-20

**Authors:** Byung-Su Kim, Sumin Kim, Eunhee Kim, Ick-Mo Chung, Sodam Jung, Yoonkyung Chang, Dong Woo Shin, Tae-Jin Song

**Affiliations:** 1 Department of Neurology, Ewha Womans University Mokdong Hospital, Ewha Womans University College of Medicine, Seoul 07985, Republic of Korea; ksm2664@hanmail.net (S.K.); tin1207@nate.com (Y.C.); dongwooshin.stroke@gmail.com (D.W.S.); 2 Department of Radiology, Ewha Womans University Mokdong Hospital, Ewha Womans University College of Medicine, Seoul 07985, Republic of Korea; kimeunheekeh@gmail.com; 3 Division of Cardiology, Department of Internal Medicine, Ewha Womans University Mokdong Hospital, Ewha Womans University College of Medicine, Seoul 07985, Republic of Korea; ickmo@ewha.ac.kr (I.-M.C.); cvdosam@gmail.com (S.J.); 4 Department of Neurology, Seoul Hospital, Ewha Womans University College of Medicine, Seoul 07804, Republic of Korea; knstar@ewha.ac.kr

**Keywords:** reversible cerebral vasoconstriction syndrome, thunderclap headache, variant angina pectoris, computed tomography, coronary angiography, spasm

## Abstract

**Background:** Reversible cerebral vasoconstriction syndrome (RCVS) is an uncommon and often underrecognized neurovascular disorder. We aimed to investigate the clinical presentations associated with extracerebral vasoconstriction in patients diagnosed with RCVS. **Methods:** In this cross-sectional study, we analyzed data from a single-center cohort of patients with RCVS in Korea. Extracerebral vasoconstriction in individuals diagnosed with RCVS was defined by the following criteria: (1) the presence of sudden and severe pain in extracerebral regions (primarily the chest or abdomen) coinciding with the onset of RCVS, (2) resolution of the pain following the administration of vasodilators, and (3) confirmation of vasoconstriction through imaging studies or, at a minimum, the exclusion of other potential causes associated with the pain. **Results:** Among the 80 eligible patients (median age, 53 years; female sex, 82.5%), 8 patients (10%) experienced extracerebral vasoconstriction. Regarding pain location, four patients reported chest pain, two reported abdominal pain, and two reported pains in both the chest and abdomen. When comparing visit route, the patients were associated with emergency department (odds ratio [OR]: 6; 95% confidence interval [CI]: 1.1–33; reference: outpatient) and inpatient consultation (OR: 25; 95% CI: 1.1–560) compared to those without extracerebral vasoconstriction. Patients with extracerebral vasoconstriction had no prior history of precipitating conditions or medication use before the onset of RCVS. The treatment response to vasodilators was excellent in all patients, and none reported neurovascular or extracerebral complications during the bout of RCVS. **Conclusions:** A co-occurrence of extracerebral vasoconstriction was not exceptionally uncommon among patients with RCVS. Our findings suggest that extracerebral vasoconstriction may be underrecognized in individuals with RCVS.

## 1. Introduction

Reversible cerebral vasoconstriction syndrome (RCVS) is an uncommon and often underrecognized neurovascular disorder caused by cerebrovascular dysregulation [1,2,3,4]. Research conducted over the past two decades has demonstrated that RCVS can be diagnosed based on the presence of thunderclap headaches (TCHs) or recurrent severe headaches, neuroimaging evidence of cerebral vasoconstriction affecting at least two different cerebral arteries, and significant resolution of vasoconstriction within 12 weeks [1,2,3,5]. Although the typical clinical course of RCVS is benign and self-limiting, some individuals may experience serious neurological complications, including ischemic stroke, subarachnoid hemorrhage, intracerebral hemorrhage, seizures, and posterior reversible encephalopathy syndrome (PRES). In this context, early recognition and accurate diagnosis of RCVS are essential for successful management [1,2,3,5,6].

Unlike the name of the disease, RCVS, a few recent case reports have described patients with RCVS who simultaneously experience coronary, renal, or colic vasoconstriction [7,8,9,10,11]. It is well established that blood pressure (BP) surges during the initial acute stage of RCVS are a common clinical manifestation resulting from sympathetic overactivity, affecting approximately half of the patients [1,12]. In addition, oxidative stress and endothelial dysfunction are believed to play key roles in the pathophysiology of RCVS. These factors are also considered significant contributors to the development of coronary vasospasm [2,13]. In this context, we hypothesized that extracerebral vasoconstriction, such as concurrent coronary vasospasm or abdominal visceral vasospasm, may occur and could be underestimated in patients with RCVS. To date, there have been no cohort-based studies reporting the prevalence of extracranial vasoconstriction in RCVS. Therefore, we aimed to investigate the presence of concurrent extracerebral vasoconstriction in patients with RCVS using cohort-based data.

## 2. Methods

### 2.1. Study Design and Patients

In this cross-sectional study, we utilized data from a prospective cohort of patients with RCVS at a single tertiary university hospital (Seoul, Korea) between April 2023 and October 2024. Each patient provided written informed consent before participation in the study. This study was approved by the local institutional review board and complied with the Declaration of Helsinki.

Comprehensive data on demographics, past medical history, substance and medication use, and clinical manifestations at the onset of the condition were collected from the patients. Appropriate laboratory workups were conducted, while cerebrospinal fluid testing was performed selectively and only when necessary [1,5]. Each patient underwent 3-Tesla magnetic resonance imaging (MRI) (Achieva; Philips Medical Systems, Best, The Netherlands, or MAGNETOM; Siemens, Germany), brain computed tomography angiography (CTA), and/or invasive catheter cerebral angiography to investigate the presence of vasoconstriction in the intracranial arterial beds and other serious intracranial and neurovascular pathologies [1,5,14]. Given the high prevalence of intracranial arterial disease among East Asians, the presence of vasoconstriction was carefully evaluated through a consensus among experienced neurologists, neurointerventionalists, and a neuroradiologist [15,16]. For a comprehensive differential diagnosis, some patients additionally underwent high-resolution vessel wall MRI, guided by clinical and neuroimaging findings [5,17]. Patients diagnosed with RCVS underwent follow-up neuroimaging between 3 and 6 months to confirm the reversibility of the cerebral vasoconstriction [1,3,5,14,17].

The diagnosis of RCVS in our cohort was meticulously established based on the following clinical and neuroradiological features: (1) clinical manifestations: TCH and severe recurrent or persistent headaches accompanied by a simultaneous BP surge; (2) common precipitants: medical conditions (such as postpartum status, head trauma, surgery, etc.), recent illnesses (including craniofacial and/or systemic infections, systemic inflammation, etc.), and substance or medication use (including illicit drugs, selective serotonin reuptake inhibitors, serotonin–norepinephrine reuptake inhibitors, and other vasoactive drugs, etc.); (3) neurovascular findings: cerebral vasoconstriction affecting at least two different intracranial arteries, with complete or considerable resolution of the vasoconstriction within three months; and (4) neurovascular complications (associated neuroimaging findings): transient focal neurological deficits, seizures, ischemic strokes, convexity subarachnoid hemorrhages, intracerebral hemorrhages, and PRES. Patients were evaluated for a diagnosis of RCVS using the aforementioned diagnostic criteria, based on a consensus among experienced neurologists and a neuroradiology specialist. Patients were started on oral nimodipine immediately upon diagnosis or clinical suspicion of RCVS [1,6,18].

### 2.2. Diagnosis of Extracerebral Vasoconstriction

Extracerebral vasoconstriction in patients diagnosed with RCVS was defined as follows: (1) the sudden onset of severe pain in extracerebral regions, primarily the chest or abdomen, coinciding with the onset of RCVS; (2) resolution of the pain with the administration of vasodilators; and (3) confirmation of extracerebral vasoconstriction through imaging studies or, at a minimum, the exclusion of other organic causes associated with the pain. Given that coronary vasospasm is typically transient, diagnosing variant angina pectoris can be particularly challenging [10,11,13,19,20]. Therefore, the primary objective of the imaging workup in this study was to exclude the presence of significant stenosis in the extracerebral arteries and to rule out other underlying conditions in the extracerebral organs that could potentially be causing the pain. If criterion 3 was not satisfied and the pain improved significantly or completely with vasodilator treatment alone, it was classified as a possible diagnosis.

### 2.3. Statistical Analysis

Continuous variables are presented as medians with interquartile ranges, while categorical variables are reported as counts and percentages. The study patients were dichotomously divided into two groups based on the presence of extracerebral vasoconstriction. We assessed factors associated with extracerebral vasoconstriction using univariable binary logistic regression analysis, calculating the odds ratio (OR) and 95% confidence interval (CI). The statistical analysis was performed using the Statistical Package for the Social Sciences (version 18.0, SPSS Inc., Chicago, IL, USA). All reported *p*-values were two tailed, and those  <0.05 were considered statistically significant.

## 3. Results

### 3.1. Study Patients

During the study period, 80 eligible patients (median age, 53 years; female, 82.5%) were enrolled in this study. The demographics, visit routes, and clinical characteristics of the study patients are presented in Table 1. Regarding the visit routes, 52 patients (65%) attended the outpatient clinic, while 26 patients (32.5%) visited the emergency department. Four patients (5%) had a history of prior bout of RCVS, 53 patients (66.3%) had preexisting migraines, and 2 patients (2.5%) reported a history of current smoking. Forty-three patients experienced BP surge during the acute stage, and 77 patients (96.2%) had multiple (or single) TCHs.

### 3.2. Patients with Extracerebral Vasoconstriction

**Figure 1:**
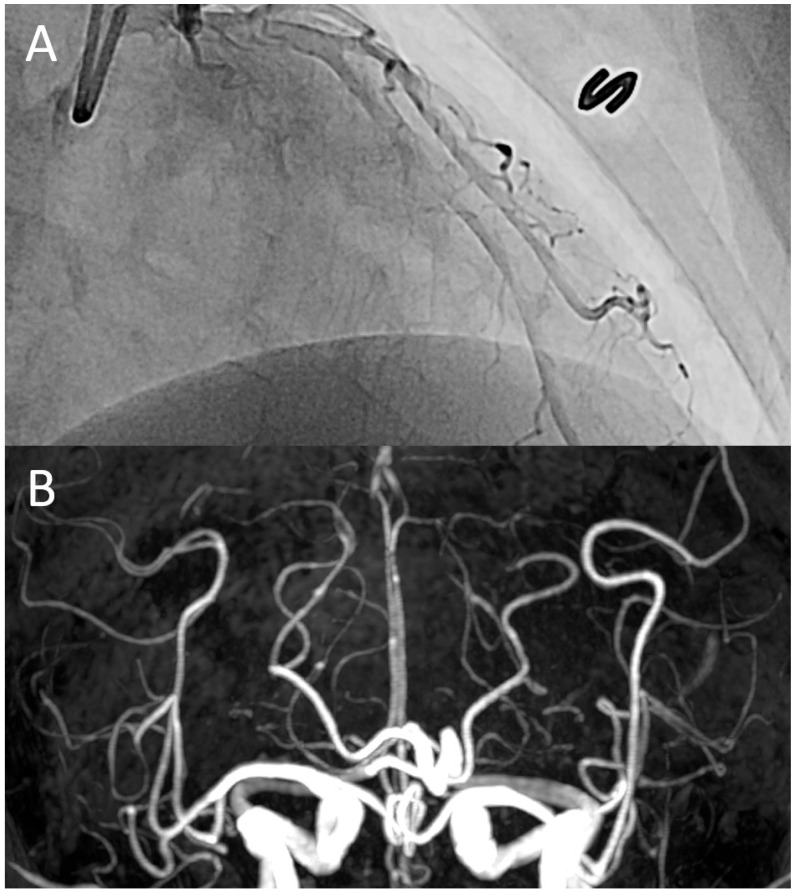
A representative case: a 52-year-old woman was admitted to the cardiology division due to recurrent episodes of sudden, severe chest pain, accompanied by thunderclap headaches and abdominal cramping. Invasive coronary angiography revealed diffuse coronary vasospasm (**A**), while time-of-flight magnetic resonance angiography demonstrated diffuse vasoconstriction in the distal segments of the intracranial arteries (**B**). These findings suggest a co-occurrence of vasoconstriction in both the heart and brain.

According to the diagnostic criteria outlined in this study, we identified eight patients with extracerebral vasoconstriction. Their characteristics, diagnostic processes, treatments, and clinical outcomes are summarized in Table 2. In the case of a 52-year-old woman, coronary vasospasm was confirmed through invasive coronary angiography (Figure 1). One patient underwent coronary computed tomography angiography (CCTA), while the other two underwent abdominal computed tomography angiography. None of these patients exhibited significant stenoses in their extracerebral arteries or other organic lesions. Four patients were diagnosed with possible extracerebral vasoconstriction, as they did not undergo a diagnostic work-up for extracerebral pain. All patients demonstrated an excellent response to vasodilator treatment. No patients reported neurovascular or extracerebral complications during the bout of RCVS.

### 3.3. Associated Factors of Extracerebral Vasoconstriction

In Table 1, we compared the demographics, visit routes, and clinical characteristics of study subjects with and without extracerebral vasoconstriction using univariable analyses. When comparing visit route, the patients were associated with emergency department (OR: 6; 95% CI: 1.1–33; reference: outpatient) and inpatient consultation (OR: 25; 95% CI: 1.1–560) compared to those without extracerebral vasoconstriction. Patients with extracerebral vasoconstriction had no documented history of precipitating conditions or medication use prior to the onset of RCVS. Additionally, the proportions of patients with a prior bout of RCVS, BP surge, and spontaneous TCH were higher among those with extracerebral vasoconstriction, although these differences did not reach statistical significance.

## 4. Discussion

The present study investigated concurrent extracerebral vasoconstriction in patients with RCVS. It was found that 10% of the patients with RCVS exhibited symptoms of extracerebral vasoconstriction, while 2.5% of these patients experienced pain in both the chest and abdomen simultaneously. Four patients with extracerebral vasoconstriction (5%) did not undergo diagnostic work-ups for their chest or abdominal pain during the acute stage; nevertheless, their extracerebral pain was effectively managed with vasodilators. Our results indicate that vasoconstriction is not limited to the head but can be multifocal in some patients with RCVS. To the best of our knowledge, this study is the first to report the prevalence of extracerebral vasoconstriction in a cohort of patients with RCVS.

Of the previous case reports, only two documented simultaneous chest and/or abdominal pain during bout of RCVS [10,11]. In contrast, other earlier studies indicated that extracerebral involvement was either incidental or subclinical [7,8,9]/ These varied observations suggest that the co-occurrence of extracerebral vasoconstriction may be more common than previously anticipated during bout of RCVS. Supporting this assumption, we identified a 46-year-old woman in our cohort who exhibited subclinical coronary vasospasm. Since she experienced only typical TCH without chest pain, she was ultimately classified as a patient without extracerebral vasoconstriction according to the diagnostic criteria of this study. She had no prior history of hypertension; however, she did experience a significant BP surge coinciding with the onset of TCH. Due to the presence of multiple old ischemic lesions affecting multivascular territories on her MRI, she underwent CCTA to assess for intracardiac and extracardiac shunts during the acute phase of RCVS. This examination revealed moderate focal stenosis in the first segment of the right coronary artery (RCA). Following the resolution of the RCVS episode, a follow-up CCTA demonstrated the disappearance of the focal stenosis in the RCA. Therefore, we can infer that the most likely etiology of her reversible focal stenosis in the RCA is vasospasm. Taken together with the earlier subclinical case reports, these findings suggest that extracerebral vasoconstriction may occur silently and frequently, often going unnoticed during bout of RCVS. Nonetheless, this hypothesis warrants further investigation in comprehensive studies.

**Figure 2:**
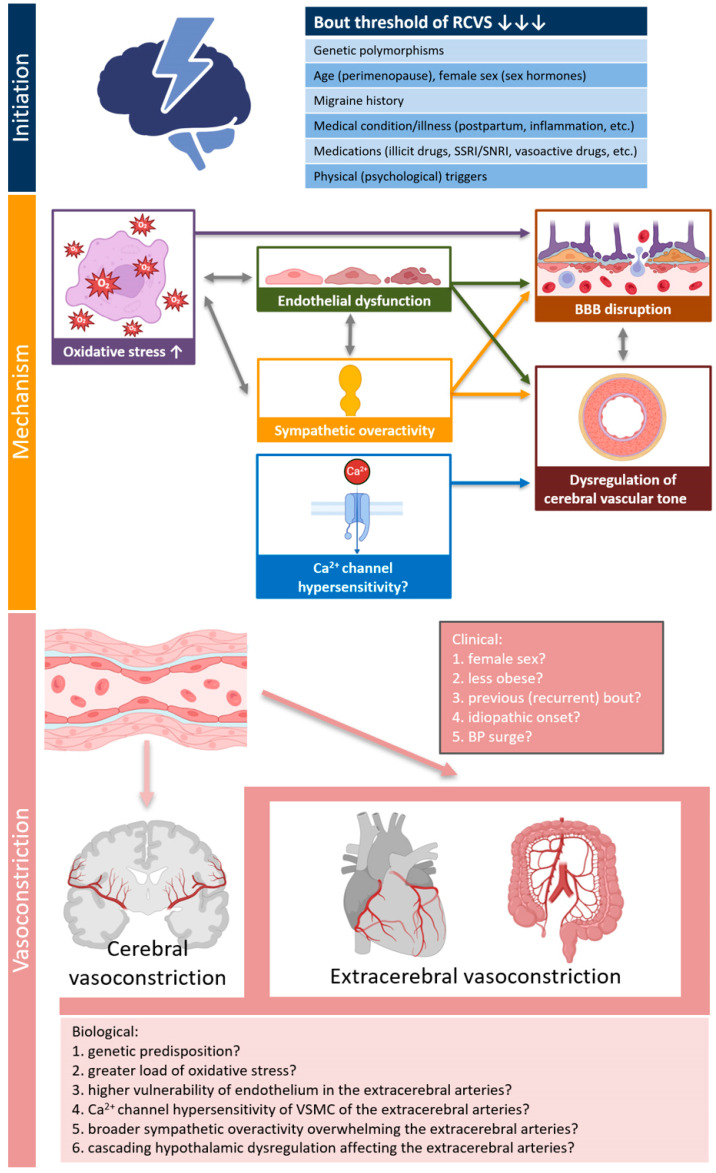
Possible explanations for concurrent extracerebral vasoconstriction in patients with RCVS. The pathophysiology model of RCVS proposed by Chen SP and Wang SJ was adopted into mechanism of RCVS [2]. Created in Biorender Kim, B. (2025) https://BioRender.com/yhjq4gy (accessed on 19 June 2025). Abbreviations: ANS, autonomic nervous system; BBB, blood-brain barrier; BP, blood pressure; RCVS, reversible cerebral vasoconstriction syndrome; VSMC, vascular smooth muscle cell.

Our initial assumption was that patients with extracerebral vasoconstriction share certain similarities with typical patients with coronary vasospasm or variant angina, particularly regarding demographic and clinical characteristics [13,19,20]. However, it is noteworthy that 7 out of 8 patients in our study were female, and all patients were non-smokers. The predominance of females in the extracerebral vasoconstriction group was slightly higher than that in the group without extracerebral vasoconstriction. Furthermore, none of the patients had any precipitating conditions or a history of drug use prior to the onset of RCVS. For these reasons, we hypothesize that extracerebral vasoconstriction may primarily be driven by greater intrinsic disease activity of RCVS in association with a predisposition of the extracerebral arteries (Figure 2). A novel pathophysiological model of RCVS proposed by Chen SP and Wang SJ suggests that oxidative stress, endothelial dysfunction, and sympathetic overactivity contribute to blood-brain barrier disruption and dysregulation of cerebral vascular tone, which play crucial roles in cerebral vasoconstriction [2]. In addition to these shared pathophysiological factors, we should also consider that genetic predisposition or hypothalamic dysregulation affecting the extracerebral arteries may contribute to the co-occurrence of extracerebral vasoconstriction during bout of RCVS [13,21,22]. However, since these biological explanations remain largely speculative, further studies are required to verify this.

In light of the clinical implications, the patients with extracerebral vasoconstriction were significantly associated with initial visits to the ED or other departments. This implies that the symptoms of extracerebral vasoconstriction are not simply associated symptoms of RCVS but can significantly influence the patient’s visit patterns and illness perception. Given that the most distinguishing feature of RCVS is TCHs or severe headaches, these results are partially consistent with a previous migraine study that emphasize the clinical significance of associated gastrointestinal symptoms [23]. Furthermore, it is important to consider that the differing visit patterns and disease perceptions of patients with extracerebral vasoconstriction may increase the likelihood of diagnostic confusion or delays in real-world clinical practice. Despite ongoing uncertainty regarding the efficacy of nimodipine in treating headaches and preventing neurovascular complications, our patients demonstrated significant resolution of both headache and extracerebral pain [1,6,18,24,25]. This finding is consistent with the standard treatment protocols for both RCVS and coronary vasospasm [1,3,13,18,19,20]. Next, the proportion of recurrent bout of RCVS was nearly three times higher in patients with extracerebral vasoconstriction. The first reported case of symptomatic coronary vasospasm involved a patient who experienced multiple stereotypical episodes of chest pain and headache over a three-year period before receiving an accurate diagnosis [10]. To summarize those findings, we can presume that patients experiencing extracerebral vasoconstriction are at an increased risk of clinical recurrence of RCVS. Considering the recurrent nature of RCVS, further studies are needed to investigate the long-term risk of clinical recurrence in patients with extracerebral vasoconstriction [26,27].

In terms of the extent of vasoconstriction, patients with extracerebral vasoconstriction may be classified as a severe subtype of RCVS. However, the study participants with extracerebral vasoconstriction did not exhibit any neurovascular or extracerebral organ complications. Despite the short-term favorable outcomes observed in this study, a prior case study involving three patients with cardiomyopathy noted that one of them experienced persistent cardiomyopathy after three months [8]. Therefore, the potential risks to the brain, heart, and visceral organs in patients with extracerebral vasoconstriction warrant further investigation in future RCVS studies.

This study has several limitations. First, our results are based on data from a single-center cohort, which raises concerns about potential selection bias. Second, all study participants were Korean. Given that coronary vasospasm is more prevalent in East Asian populations, the risk of co-occurring extracerebral vasoconstriction in RCVS may vary among different racial groups [28]. Third, the results from the logistic regression analyses should be interpreted with caution, because of a lack of temporal relationship. Fourth, the diagnostic criteria for extracerebral vasoconstriction in this study heavily relied on clinical features, which may result in a significant risk of misclassification bias. Consequently, the findings of this study should be validated in future research that employs a strategy of extensive and comprehensive imaging workups. Fifth, the small sample size of the extracerebral vasoconstriction group may considerably limit the statistical power of the analysis. Therefore, the results of the statistical analysis in this study should be interpreted with these limitations in mind. Lastly, our results demonstrated good efficacy of nimodipine in patients with extracerebral vasoconstriction; however, there is ongoing debate regarding the efficacy of nimodipine in certain patients with RCVS. Therefore, the therapeutic efficacy of nimodipine should be validated in future studies.

## 5. Conclusions

In conclusion, the co-occurrence of extracerebral vasoconstriction was not exceptionally uncommon among patients with RCVS. Awareness of extracerebral vasoconstriction in RCVS has been evolving since the previous case reports leading up to this study. Our findings suggest that extracerebral vasoconstriction and associated clinical manifestations may be underrecognized in individuals with RCVS.

## Figures and Tables

**Table 1:** Demographics, visit route, and clinical characteristics with univariable odds ratios for concurrent extracerebral vasoconstriction in patients with reversible cerebral vasoconstriction syndrome.

		Extracerebral Vasoconstriction			
	Total	Absent	Present		
	(*n* = 80)	(*n* = 72)	(*n* = 8)	OR (95% CI)	*p*
Demographics					
Age, yr	53 (44–59)	53 (44–59)	55 (40–63)	1.00 (0.94–1.05)	0.987
Female sex, no. (%)	66 (82.5)	59 (81.9)	7 (87.5)	1.54 (0.17–13.63)	0.697
BMI, kg/m^2^	23.4 (21–25.6)	23.5 (21.1–25.9)	21.7 (20–24.5)	0.88 (0.7–1.12)	0.32
Visit route, no. (%)					
Outpatient	52 (65)	50 (69.4)	2 (25)	reference	
Emergency department	26 (32.5)	21 (29.2)	5 (62.5)	5.95 (1.06–33.14)	0.042
Inpatient consultation	2 (2.5)	1 (1.4)	1 (12.5)	25 (1.11–561.28)	0.043
Past history, no. (%)					
Previous bout of RCVS	4 (5)	3 (4.2)	1 (12.5)	3.28 (0.3–35.96)	0.33
Preexisting migraine	53 (66.3)	48 (66.7)	5 (62.5)	0.83 (0.18–3.78)	0.813
Hypertension	10 (12.5)	9 (12.5)	1 (12.5)	1 (0.11–9.1)	>0.999
Diabetes	4 (5)	4 (5.6)	0 (0)	NA	
Dyslipidemia	16 (20)	16 (22.2)	0 (0)	NA	
Current smoking	2 (2.5)	2 (2.8)	0 (0)	NA	
Precipitating conditions, no. (%)					
Medical conditions and illness	10 (12.5)	10 (13.9)	0 (0)	NA	
Head trauma	1 (1.3)	1 (1.4)	0 (0)	NA	
URI or other ENT infection	6 (7.5)	6 (8.3)	0 (0)	NA	
Any other infection	3 (3.8)	3 (4.2)	0 (0)	NA	
Other medical illness	1 (1.3)	1 (1.4)	0 (0)	NA	
Medications before clinical onset	6 (7.5)	6 (8.3)	0 (0)	NA	
SSRI or SNRI	1 (1.3)	1 (1.4)	0 (0)	NA	
Triptans	1 (1.3)	1 (1.4)	0 (0)	NA	
Nasal decongestants	1 (1.3)	1 (1.4)	0 (0)	NA	
Steroids	2 (2.5)	2 (2.8)	0 (0)	NA	
Immunosuppressant	2 (2.5)	2 (2.8)	0 (0)	NA	
Clinical manifestation					
BP status at acute stage					
BP surge, no. (%)	43 (53.8)	38 (52.8)	5 (62.5)	1.49 (0.33–6.71)	0.603
Systolic BP, mmHg	141 (122–153)	140 (122–152)	145 (118–194)	1.01 (0.98–1.04)	0.279
Diastolic BP, mmHg	83 (74–90)	83 (75–90)	81 (72–98)	1.01 (0.96–1.07)	0.501
TCH presentation, no. (%)					
Number					
Multiple	66 (82.5)	58 (80.6)	8 (100)	NA	
Single	11 (13.8)	11 (15.3)	0 (0)		
None	3 (3.8)	3 (4.2)	0 (0)		
Triggers					
Sexual activity	10 (12.5)	9 (12.5)	1 (12.5)	1 (0.11–9.1)	>0.999
Exertional exercise	18 (22.5)	15 (20.8)	3 (37.5)	2.28 (0.48–10.63)	0.294
Valsalva maneuverers	23(28.8)	21 (29.2)	2 (25)	0.81 (0.15–4.33)	0.805
Emotional stress	15 (18.8)	12 (16.7)	3 (37.5)	3 (0.63–14.27)	0.167
Bathing or showering	5 (6.3)	4 (5.6)	1 (12.5)	2.42 (0.23–24.84)	0.455
Head motion	12 (15)	10 (13.9)	2 (25)	2.06 (0.36–11.7)	0.412
None (spontaneous onset)	46 (57.5)	39 (54.2)	7 (87.5)	5.92 (0.69–50.64)	0.104
Neurovascular complications, no. (%)					
Focal neurological deficits	5 (6.3)	5 (6.9)	0 (0)	NA	
Intracranial artery dissection	4 (5)	4(5.6)	0 (0)	NA	
Intracranial hemorrhage	1 (1.3)	1 (1.4)	0 (0)	NA	
Seizure	3 (3.8)	3 (4.2)	0 (0)	NA	
PRES	1 (1.3)	1 (1.4)	0 (0)	NA	

Data are presented with interquartile range or number (percentage). Abbreviations: BMI, body mass index; BP, blood pressure; CI, confidence interval; ENT, ear-nose-throat; NA, not applicable; OR, odds ratio; PRES, posterior reversible encephalopathy syndrome; SNRI, serotonin–norepinephrine reuptake inhibitor; SSRI, selective serotonin reuptake inhibitor; TCH, thunderclap headache; URI, upper respiratory infection; RCVS, reversible cerebral vasoconstriction syndrome.

**Table 2:** Summary on demographics, clinical characteristics, and clinical outcome of the cases with concurrent extracerebral vasoconstriction.

				Extracerebral Vasoconstriction					Clinical Outcome	
	Sex /Age	Visit Route	Past History /Smoking Status	Pain Location	Pain Resolution by Vasodilators	Diagnostic Work-Up	Confirmation by Imaging Findings	Exclusion of Other Pain Sources	Neurovascular Complications	Extracerebral Organ Complications
1	F/52	Inpatient consultation	None/never	Chest, abdomen	O (diltiazem PO, nimodipine PO)	iCAG, abdominal ultrasound, EGD	O	O	None	None
2	F/43	Outpatient	None/never	Chest	O (nimodipine PO)	Not done	X	X	None	None
3	F/63	ED	None/never	Chest	O (nimodipine PO)	CCTA	X	O	None	None
4	M/40	ED	HTN/never	Chest	O (nimodipine PO)	Not done	X	X	None	None
5	F/22	Outpatient	None/never	Chest	O (nimodipine PO)	Not done	X	X	None	None
6	F/59	ED	None/never	Chest, abdomen	O (nimodipine PO)	CCTA, abdominal CTA	X	O	None	None
7	F/64	ED	None/never	Abdomen	O (nimodipine PO)	Abdominal CTA	X	O	None	None
8	F/63	ED	None/never	Abdomen	O (nimodipine PO)	Not done	X	X	None	None

Abbreviations: CCTA, coronary computed tomography angiography; CTA, computed tomography angiography; ED, emergency department; EGD, esophagogastroduodenoscopy; HTN, hypertension; iCAG, invasive coronary angiography.

## Data Availability

The data used in the present study are available from the corresponding author on reasonable request.

## Abbreviations

The following abbreviations are used in this manuscript:

RCVS	Reversible cerebral vasoconstriction syndrome
TCH	Thunderclap headache
BP	Blood pressure
MRI	Magnetic resonance imaging
CTA	Computed tomography angiography
PRES	Posterior reversible encephalopathy syndrome
OR	Odds ratio
CI	Confidence interval
CCTA	Coronary computed tomography angiography
RCA	Right coronary artery
